# Id2 exerts tumor suppressor properties in lung cancer through its effects on cancer cell invasion and migration

**DOI:** 10.3389/fonc.2022.801300

**Published:** 2022-08-02

**Authors:** Jian-Ting Chen, Yuan-Ling Hsu, Yi-Chiung Hsu, Yi-Hsin Tseng, Ming-Han Liu, Chia-Wei Weng, Ching-Hao Lin, Szu-Hua Pan, Jeremy J.W. Chen, Chi-Chung Wang

**Affiliations:** ^1^ Graduate Institute of Biomedical and Pharmaceutical Science, Fu Jen Catholic University, New Taipei City, Taiwan; ^2^ Graduate Institute of Medical Genomics and Proteomics, National Taiwan University, Taipei, Taiwan; ^3^ Department of Biomedical Sciences and Engineering, National Central University, Taoyuan, Taiwan; ^4^ Institute of Biomedical Sciences, National Chung Hsing University, Taichung, Taiwan; ^5^ Department of Nephrology, Sijhih Cathay General Hospital, New Taipei City, Taiwan; ^6^ Institute of Molecular Biology, National Chung Hsing University, Taichung, Taiwan

**Keywords:** FAK, inhibitor of DNA binding protein 2, lung adenocarcinoma, metastasis, twist

## Abstract

**Background:**

Despite advances in prognosis and treatment of lung adenocarcinoma (LADC), a notable non–small cell lung cancer subtype, patient outcomes are still unsatisfactory. New insight on novel therapeutic strategies for LADC may be gained from a more comprehensive understanding of cancer progression mechanisms. Such strategies could reduce the mortality and morbidity of patients with LADC. In our previous study, we performed cDNA microarray screening and found an inverse relationship between inhibitor of DNA binding 2 (Id2) expression levels and the invasiveness of LADC cells.

**Materials and Methods:**

To identify the functional roles of Id2 and its action mechanisms in LADC progression, we successfully established several Id2-overexpressing and Id2-silenced LADC cell clones. Subsequently, we examined *in vitro* the effects exerted by Id2 on cell morphology, proliferation, colony formation, invasive, and migratory activities and examined *in vivo* those exerted by Id2 on cell metastasis. The mechanisms underlying the action of Id2 were investigated using RNA-seq and pathway analyses. Furthermore, the correlations of Id2 with its target gene expression and clinical outcomes were calculated.

**Results:**

Our data revealed that Id2 overexpression could inhibit LADC cells’ migratory, invasive, proliferation, and colony formation capabilities. Silencing Id2 expression in LADC cells reversed the aforementioned inhibitory effects, and knockdown of Id2 increased LADC cells’ metastatic abilities *in vivo*. Bioinformatics analysis revealed that these effects of Id2 on cancer progression might be regulated by focal adhesion kinase (FAK) signaling and CD44/Twist expression. Furthermore, in online clinical database analysis, patients with LADC whose Id2 expression levels were high and FAK/Twist expression levels were low had superior clinical outcomes.

**Conclusion**

Our data indicate that the Id2 gene may act as a metastasis suppressor and provide new insights into LADC progression and therapy.

## Introduction

Among the most common cancers globally, lung cancer constitutes the foremost cause of cancer mortality. Within the United States, in 2022, lung cancer accounted for an estimated 12.3% of new diagnoses of cancer and >21% of all deaths due to cancer (1). Patients diagnosed as having lung cancer exhibit unsatisfactory outcomes, and such outcomes are generally ascribed to difficulties in early detection; advanced-stage lung cancer is diagnosed in >75% of patients (2). Despite the potential of low-dose computed tomography for earlier diagnosis, this modality remains challenging for the general population. For patients who have received a lung cancer diagnosis, overall 5-year survival is still less than 19% (1). The most crucial factor for patient survival is metastasis; however, the molecular aspects of metastasis have not been completely defined (3). Non–small cell lung cancer (NSCLC) constitutes 85% of all cases of lung cancer; in addition, nearly 50% of NSCLC cases are lung adenocarcinoma (LADC) (4). Although numerous studies have been conducted on LADC progression, the molecular mechanisms of this type of cancer are not clearly understood (5).

We previously used cDNA microarray analysis to screen a panel of LADC cells with different invasive capacities to identify potential genes associated with LADC metastasis (6). We successfully identified several candidate invasive suppressor genes from the aforementioned cell line model and demonstrated their roles and the corresponding molecular mechanisms in the NSCLC cell metastatic process (7,8). A differentially expressed invasion-associated gene selected in the cDNA microarray assay was inhibitor of DNA binding 2 (Id2), the expression of which had a negative correlation with the invasiveness of the cell lines. The inhibitor of DNA binding (Id) protein family is reportedly involved in numerous developmental and cellular processes as well as in carcinogenesis (9–11). In this protein family, four members (Id1–4) have been found in mammals (12). Because they lack the basic DNA-binding domain, Id proteins can heterodimerize with basic helix–loop–helix (bHLH) transcription factors and then inhibit their binding to DNA (13,14).

Recently, numerous studies have demonstrated that the Id protein family plays roles in cell cycle control, cancer development, angiogenesis, and apoptosis in a variety of human cancers (15–18). Relevant studies have indicated that Id2 might be dysregulated in tumor progression in several cancer types, such as prostate, breast, colon and rectal, head and neck, and central nervous system cancers (19–23). However, Id2 was reported to play different roles in different cancer cells (24). In prostate cancer cells, constitutive expression of Id2 could promote aggressive phenotypes (25). By contrast, Id2 expression had a negative correlation with a poorly differentiated breast cancer phenotype (26). Additionally, Id2 was found to suppress prometastatic transcriptional programs in human melanoma cells (27). Research executed recently has demonstrated that Id2 might serve as a prognostic marker for patients diagnosed as having small-cell lung cancer or having poorly differentiated tumors in NSCLC (22,28). Because the roles of Id2 differ depending on the cancer type, the determination of how Id2 affects LADC progression and metastasis is an urgent need.

In the present study, we successfully established several Id2-overexpressing and Id2-silenced LADC cell lines. Then, we clarified the roles of Id2 in LADC metastasis and tumor progression *in vitro* and *in vivo*. We demonstrated that Id2 overexpression could inhibit LADC cells’ migratory, invasive, proliferation, and colony-formation capabilities. Silencing Id2 expression in LADC cells could also reverse the aforementioned inhibitory effects. Furthermore, *in vivo* metastasis assays demonstrated that nodules significantly increased in number in mice that received tail-vein injections of Id2-silenced cells compared with those that received control cells. RNA sequencing and pathway analysis suggested that Id2 serves as an invasion suppressor gene in LADC and is highly influential in the progression and metastasis of LADC through CD44/Twist axis and focal adhesion pathway regulation. In addition, LADC patients with high Id2 expression levels have superior clinical outcomes. All these efforts extend our understanding of how the Id2 gene affects LADC progression and metastasis; this understanding is critical for the development of LADC treatment strategies in the future for clinical application.

## Materials and methods

### Cell line and culture conditions

We previously established the human CL1-5 and CL1-0 cell lines of LADC (of which CL1-5 has higher invasive capabilities) (6). We cultured all mentioned cell lines, including NCI-H322M and NCI-H1299 (ATCC CRL-5803), in RPMI-1640 medium (Invitrogen, Inc., Waltham, MA, USA) containing 10% heat-inactivated fetal bovine serum (FBS; Invitrogen) and 1% penicillin-streptomycin (both from Invitrogen) and kept the culture in a 5% CO_2_ humidified atmosphere at 37°C. For subculturing, 0.1% trypsin–0.05% EDTA (Sigma-Aldrich, St. Louis, MO, USA) was employed to dissociate the cells from the culture plates. Cells were subcultured every 3 days up to passage 10.

### Plasmid formation and stable cell establishment

Using TRIzol reagent (Invitrogen), we isolated total RNA from CL1-0 cells for full-length Id2 cDNA cloning. Moreover, we applied SuperScript II reverse transcriptase (Invitrogen) and oligo-dT primers to execute the reverse transcription of first-strand cDNA. Id2 coding region amplification was executed through a polymerase chain reaction (PCR). Into the pcDNA3.1-V5-His TOPO vector (Invitrogen; pcDNA3.1-Id2), the amplified product was subsequently cloned. Lipofectamine 2000 transfection reagents (Invitrogen) were used to transfect purified pcDNA3.1-Id2 or pcDNA3.1 plasmid into CL1-5 cells in accordance with the manufacturer protocol to further establish the vector control or CL1-5/Id2-overexpressing stable cells. Stably overexpressed transfectants were selected using Geneticin (G418; Merck, Darmstadt, Germany). The shNC negative control and shID2 (shID2-284 and shID2-528) plasmids were purchased from Biotools Co., Ltd. (New Taipei, Taiwan). To establish CL1-0/Id2-knockdown or negative control stable cells, Lipofectamine 2000 reagents were used to transfect shID2 or shNC plasmids into CL1-0 cells, and stable cell clones were isolated through Geneticin selection.

### Real-time quantitative reverse transcription PCR

The cells were homogenized, and TRIzol reagent (Invitrogen) was applied to execute total RNA extraction from the tested cell lines in accordance with the manufacturer protocol. To examine the Id2 or candidate gene mRNA expression levels in the tested cell lines, reverse transcription (RT)-PCR was implemented with SYBR Green as described previously (4). All primers used for SYBR Green real-time RT-PCR were listed in **Supplementary Table 1**.

### Western blot analysis

Through Western blot analysis, Id2 expression levels were examined after Id2 expression plasmid transfection or knockdown in the tested cells. The detailed procedures were performed as previously described (7). Primary antibodies specifically against Id2 (ab166708; 1:1,000) were purchased from Abcam. For gel loading, the internal control comprised an anti-glyceraldehyde-3-phosphate dehydrogenase (anti-GAPDH, GT239; 1:10,000) antibody (GeneTex, Inc. Irvine, CA, USA). The membranes were first incubated with the primary antibodies. Subsequently, Tris-buffered saline and Tween 20 solution (Abcam) was applied to wash the membranes three times, after which they were incubated with horseradish peroxidase–conjugated secondary antibodies (Santa Cruz Biotechnology, Inc., Dallas, TX, USA). Signals were detected using enhanced chemiluminescence (ECL, GE Healthcare, Piscataway, NJ, USA).

### Immunofluorescence staining

We cultured the tested cells on 12-mm glass coverslips, followed by fixing them for 15 min in 4% paraformaldehyde (Thermo Fisher Scientific, Inc., Carlsbad, CA, USA), permeabilizing them, and staining them. The cellular actin filaments were stained using TRITC-conjugated phalloidin (Sigma-Aldrich). The detailed procedures were performed as previously described (8). A fluorescent microscope (Leica DM2000, Germany) was used to examine and photograph the cells.

### Cell proliferation assay

Cell proliferation capabilities were evaluated through trypan blue exclusion and thiazolyl blue tetrazolium bromide (MTT) assays. The cells were seeded for 24, 48, 72, and 96 h at 5 × 10^4^ cells/well in the trypan blue exclusion assay. Subsequently, the cells were counted with trypan blue solution (Sigma-Aldrich). A hemocytometer was used to count the living cells under an inverted light microscope. The MTT assay was executed by seeding the cells at 3 × 10^3^ cells/well into 96-well plates. After various culturing durations, the MTT assay was performed to examine cell proliferation activity in accordance with manufacturer protocols (Sigma-Aldrich).

### Colony formation assay

The cells’ anchorage-dependent colony formation activity was determined by seeding them at 300 cells/well into six-well plates in RPMI-1640 medium. Every 2–3 days, the culture medium was changed. After 7–10 days, the medium was removed; subsequently, the cells were fixed and stained with 0.05% crystal violet (Sigma-Aldrich). Furthermore, the cells’ anchorage-independent growth was determined by precoating the six-well plates with 0.7% agarose in RPMI-1640 and then seeding the cells at 3,000 cells/well in 0.35% agarose/RPMI-1640 with 10% FBS. Every 2–3 days, the culture medium was changed. The plates were subjected to a 4–5-week incubation process executed in 5% CO_2_ at 37°C, followed by staining with crystal violet for 30-60 min. An inverted light microscope was employed to count colonies larger than 0.8 mm. Two independent experiments in triplicate were performed for the colony formation assays.

### Cell migration and invasion assays

The migratory capabilities of the tested cells were evaluated using the previously described wound healing approach (29). A light microscope was used to count cells that migrated into the cell-free zone at indicated times (T = 0 and 8 h). A Transwell assay with Matrigel-coated transwell filters (BD Biosciences, Franklin Lakes, NJ, USA) and modified Boyden chambers (pore size: 8 μm; Corning Costar, Cambridge, MA, USA) was used to assess cell invasiveness as previously described (29). After 18 h of incubation at 37°C, the cells were fixed for 10 min in methanol (Sigma-Aldrich) and then stained at room temperature for 30 min with 10% Gemisa Stain solution (Sigma-Aldrich). A light microscope (200× magnification) was employed to quantify the cells detected to be attached to the lower surfaces of the polycarbonate filters. All aforementioned experiments were executed in triplicate.

### *In vivo* metastasis assay

A previously described procedure for the *in vivo* tail-vein metastasis assay was used (8). In brief, 6-week-old SCID mice (provided by the National Laboratory Animal Center, Taiwan, n = 5 per group) received lateral tail-vein injections of a single-cell suspension of 10^6^ CL1-0/vector (CL1-0/shNC) or CL1-0/Id2-knckdown (CL1-0/shID2) cells in 0.1 mL of PBS. Ten weeks postinjection, the lungs of the mice were removed after the mice were sacrificed through carbon dioxide anesthesia. A mouse was placed in a 1-liter volume chamber and used a CO_2_ flow rate of 0.5 liters per minute. The method was following the suggestions of AVMA Guidelines for the Euthanasia of Animals (2020 Edition) (30). Subsequently, the derived lungs were weighed before being fixed in 10% formalin (Thermo Fisher Scientific). A dissecting microscope was employed to count the number of metastatic nodules. Hematoxylin and eosin (H&E) staining was then executed on 4-μm-thick sections of embedded tissues for histological analysis. The Laboratory Animal Center, National Taiwan University College of Medicine, approved all *in vivo* experiments (IACUC number: 20140034).

### RNA sequencing analysis

The sequencing library was prepared using purified RNA with a TruSeq Stranded mRNA Library Prep Kit (Illumina, San Diego, CA, USA) in accordance with the manufacturer instructions. In brief, oligo(dT)-coupled magnetic beads were used to purify 1 μg of total RNA into an mRNA sample. At a high temperature, the sample was then fragmented into pieces. Then, random primers and reverse transcriptase were used for first-strand cDNA synthesis. After the generation of double-stranded cDNA and adenylation of the 3′ ends of the DNA fragments, an AMPure XP system (Beckman Coulter, Beverly, MA, USA) was used to ligate and purify the adaptors. To execute library quality assessments, the Agilent Real-Time PCR and Bioanalyzer 2100 systems were employed. Subsequently, an Illumina HiSeq 4000 platform was used to sequence the qualified libraries, for which Genomics, BioSci & Tech Co. (New Taipei City, Taiwan) generated 150-bp paired-end reads. RNAseq data are publicly available on NCBI’s Sequence Read Archive (SRA) database (Bio-project: PRJNA778932).

### Bioinformatics analysis

The Trimmomatic program (version 0.39) was used to remove the low-quality bases and sequences from the adapters in the raw data (31). In addition, Bowtie2 (version 2.3.4.1) was used to align the filtered reads with the reference genomes as previously described (32). Moreover, the user-friendly software RSEM (version 1.2.28) was employed to quantify the transcript abundance (33). For the identification of differentially expressed genes, EBSeq (version 1.16.0) was executed (34). Gene Ontology term and Kyoto Encyclopedia of Genes and Genomes (KEGG) pathway enrichment analyses among gene clusters were implemented in an R package called ClusterProfiler (version 3.6.0) (35–37).

### Statistical analysis

The data of all experiments (which were executed in triplicate) were subjected to analysis of variance (Microsoft Excel) for significance testing. Results are presented as mean ± standard deviation (SD). For survival analysis, The Cancer Genome Atlas (TCGA) was used to derive Id2, FAK, and Twist mRNA profiles in 502 patients with LADC. Disease-free survival and mRNA expression data were downloaded from the cBio portal (38). The summed target-gene mRNA expression levels represented the risk score of a patient. Patients were classified into a group of high or low expression, with the median risk score being the threshold value. Both the high- and the low-expression groups’ survival curves were analyzed and compared through Kaplan–Meier analysis and a log-rank test, respectively. Two-sided statistical tests were performed, and significance was indicated by *P* < 0.05.

## Results

### Association of Id2 expression with cancer cell invasion

**Figure 1:**
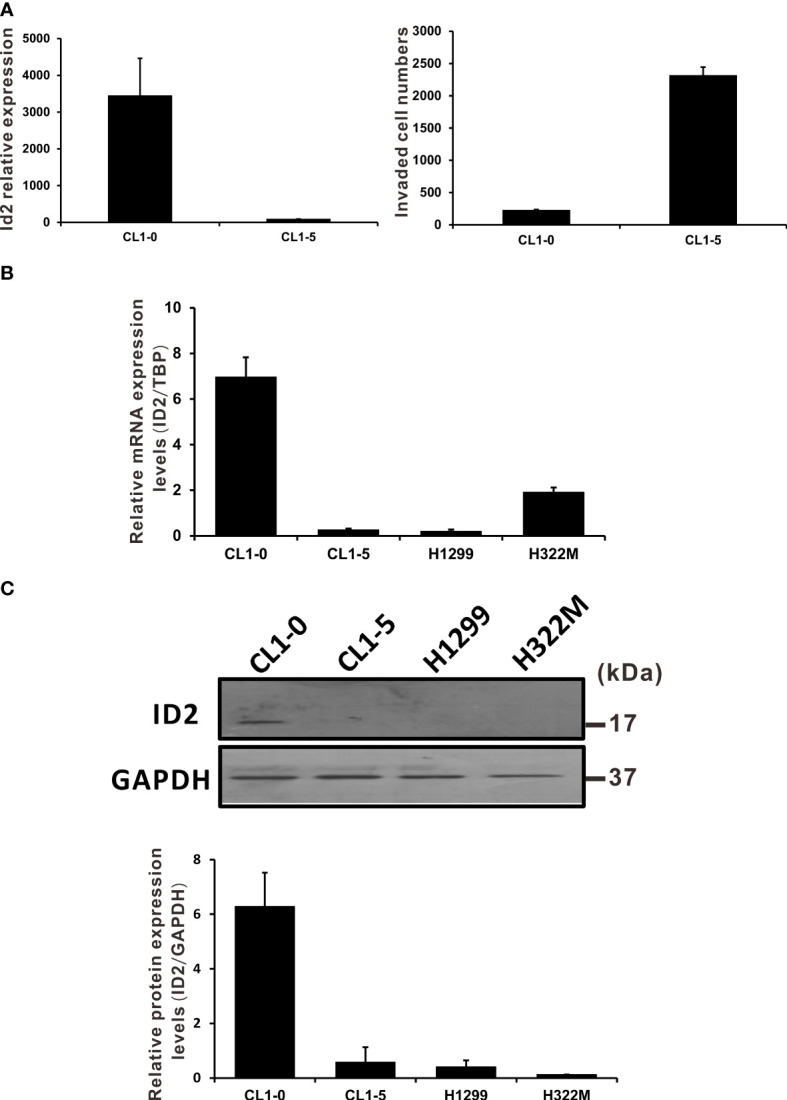
NSCLC cell invasion was negatively correlated with Id2 expression. **(A)** Id2 mRNA expression levels in a cell line model of LADC (microarray analysis, left panel). CL1-5 cells exhibited greater invasive abilities than did CL1-0 cells (right panel). **(B)** Id2 mRNA expression evaluated through qRT-PCR and **(C)** protein levels evaluated in Western blot analysis in different NSCLC cells. Real-time RT-PCR assays used TATA-binding protein as an internal control. All experiments were executed in triplicate. Loading control in Western blots: GAPDH.

We previously identified associations between the expression levels of several hundred genes and the invasive capabilities of lung cancer cells (6). We established a panel of human LADC cell lines exhibiting various invasive abilities and screened it using cDNA microarray analysis. By analyzing the genes considered in this study, we found the microarray signal of Id2 to be inversely associated with cell invasive ability (**Figure 1A**). To confirm the microarray analysis results, we performed qRT-PCR analysis, wherein we measured Id2 mRNA expression levels in various NSCLC cell lines. The qRT-PCR results indicated higher Id2 mRNA expression in the CL1-0 cells (which are less invasive) compared with that in the CL1-5 cells (which are highly invasive; **Figure 1B**). In addition, Id2 expression in the other tested cell lines (e.g., H1299 and H322M) was low. Protein expression levels of Id2 were also higher in the CL1-0 cells than in the other lung cancer cells (**Figure 1C**).

### Suppression of LADC cell progression *in vitro* through Id2 overexpression

**Figure 2:**
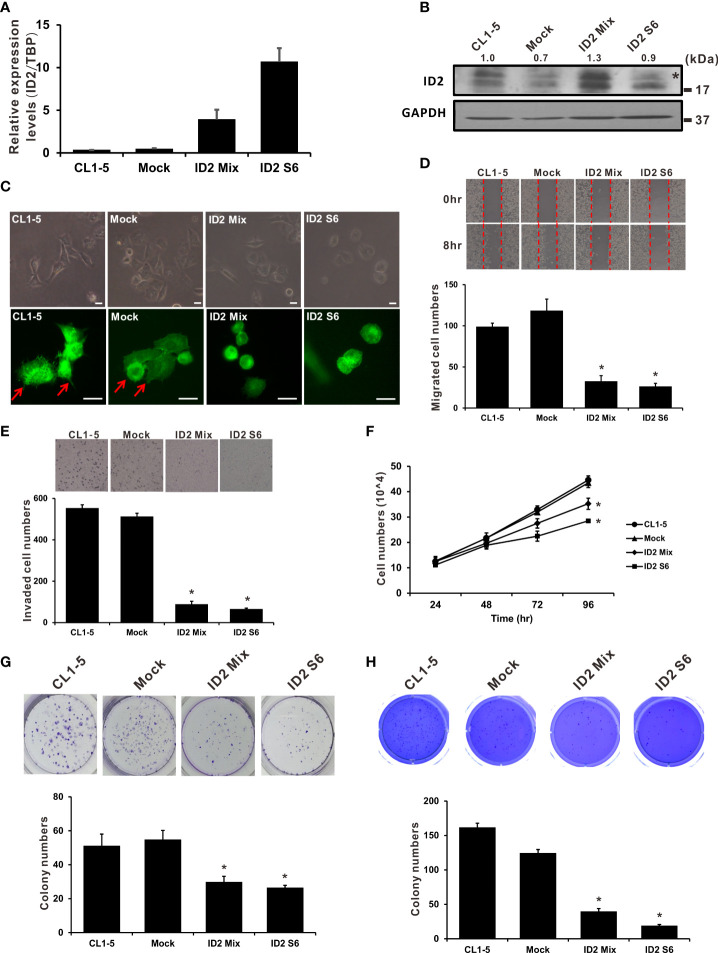
Overexpression of Id2 suppressed highly invasive LADC cells’ aggressive properties. **(A)** mRNA expression of Id2 in transfected CL1-5 cells measured using qRT-PCR. The control cells (Mock) were pcDNA3.1 vector transfectants. Internal control: TBP. **(B)** Expression of Id2 protein in the transfectants, measured through Western blot analysis with an antibody against Id2; loading control: GAPDH. *, non-specific band. **(C)** Representative images of stably expressing Id2 or control vector cells; morphology and immunofluorescence staining of endogenous F-actin. Red arrows indicate the filopodia of cells; scale bar, 20 μm. **(D)** Scratch wound healing assays for assessing Id2 transfectant cell migratory ability. Eight hours after wound affliction, the cells migrating to the cell-free zone were counted. Data are presented as mean ± SD and represent three independent experiments. *P < 0.05 versus mock control. **(E)** Transwell assays were used to evaluate mock, Id2, and CL1-5 transfectant invasiveness in three independent experiments. *P < 0.05 versus mock control. **(F)** Trypan blue exclusion assay for examination of mock, Id2, and CL1-5 transfectant proliferation activity. *P < 0.05 versus mock control. Assays of **(G)** anchorage-dependent as well as **(H)** anchorage-independent colony formation were also executed in mock, Id2, and CL1-5 transfectants. *P < 0.05 versus the mock control.

We established stably constitutive Id2-expressing CL1-5 cell clones to elucidate the influence of Id2 expression on LADC progression. We executed qRT-PCR and Western blot analyses to determine Id2 mRNA and protein expression, respectively, in the stable cell lines (**Figures 2A, B**). A single clone (ID2 S6) and a mixed clone (ID2 Mix) that highly expressed Id2 in the CL1-5 cells were isolated for further study. To investigate whether the cellular morphology was changed after Id2 overexpression, the cellular F-actin filaments were stained using TRITC-conjugated phalloidin. The CL1-5/Id2-overexpressing stable cells exhibited an epithelial-like morphology compared with the parental CL1-5 and vector control (Mock) cells, which showed the mesenchymal-like morphology with more filopodia (**Figure 2C** and **Supplementary Figure 1**). In the wound healing assay, the migratory abilities of the highly expressed Id2 transfectants (ID2 S6 and ID2 Mix) were considerably diminished relative to the mock control cells’ migratory abilities (**Figure 2D**; P < 0.05). Furthermore, the invasive capabilities of the ID2 S6 and ID2 Mix cells were significantly inferior to those of the CL1-5 and mock control cells (**Figure 2E**; *P* < 0.05). We performed a cell proliferation essay to exclude the possibility that Id2 inhibited LADC cell migration and invasion by blocking proliferation. **Figure 2F** indicates that only after 72 h did the Id2 transfectants significantly diminish proliferation activity relative to the CL1-5 and mock control cells (P < 0.05). The significantly reduced Id2 transfectant colony formation compared with the mock control cells indicates that Id2 inhibits anchorage-dependent and anchorage-independent growth (**Figures 2G, H**; *P* < 0.05).

### Rescue of LADC cell aggressiveness by Id2 knockdown

**Figure 3:**
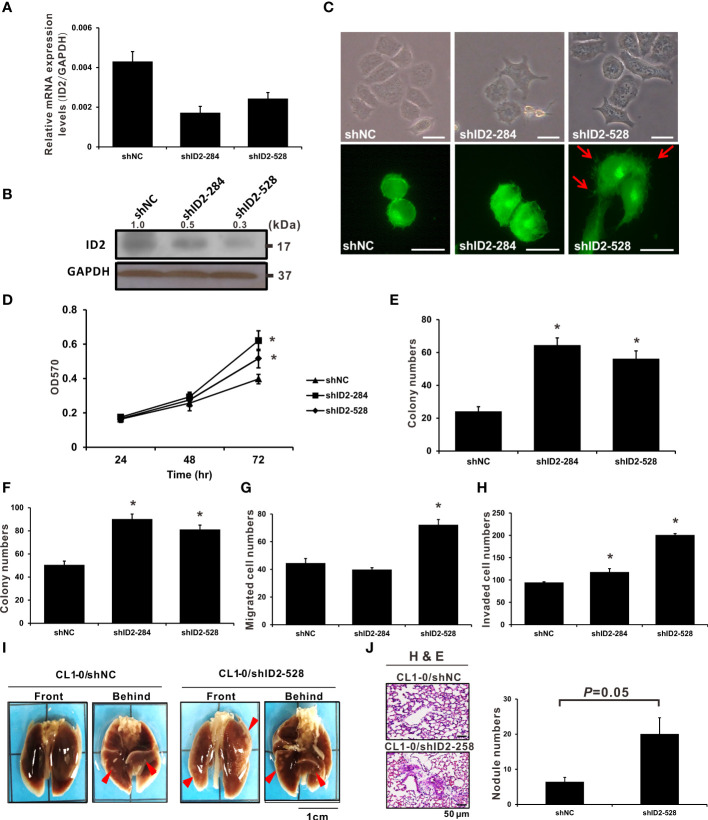
Knockdown of Id2 expression promoted *in vitro* and *in vivo* lung adenocarcinoma cell aggressiveness. **(A)** qRT-PCR and **(B)** Western blot analysis executed to determine mRNA and protein expression levels, respectively, of Id2 in CL1-0/Id2-knockdown (shID2-284 and shID2-528) and control (shNC) transfected cells. Internal or loading control: GAPDH. **(C)** Representative Id2-knockdown or shNC cell images revealing their morphology and endogenous F-actin immunofluorescence staining. Red arrows indicate the filopodia of cells; scale bar, 20 μm. **(D)** MTT assay was performed to assess Id2-knockdown and shNC cell proliferation. *P < 0.05 versus shNC control. **(E)** anchorage-dependent and **(F)** anchorage-independent colony formation assays were also performed in Id2-knockdown and shNC cells. *P < 0.05 versus shNC control. **(G)** Migratory and **(H)** invasive capabilities of Id2-knockdown and shNC cells, respectively. *P < 0.05 versus shNC control. **(I)** *In vivo* tail-vein metastasis assay of Id2 expression in cancer metastasis. Red arrows indicate tumor nodules; scale bar, 1 cm. **(J)** Histological confirmation through H&E staining. Five mice per group were selected for metastatic tumor nodule counting (*P* = 0.05); scale bar, 50 μm.

To further investigate how Id2 downregulation affects LADC cell function, we analyzed CL1-0 cells with stably transfected shNC (control) and two specific Id2 shRNA plasmids (shID2-284 and shID2-528). **Figures 3A, B** indicates that the Id2 mRNA and protein expression levels in these Id2-knockdown cells decreased substantially relative to those in the shNC-transfected CL1-0 cells. shID2-528 is more effective than shID2-284 in decreasing the ID2 protein expression in lung cancer cells. The morphology of the Id2-knockdown cells was examined, revealing elongated, spindly, and dispersed CL1-0/shID2-528 cells; relative to the control cells, this morphology was more similar to mesenchymal cells (**Figure 3C**). Therefore, the expression levels of numerous epithelial and mesenchymal markers were examined by Western blot analysis. As shown in **Supplementary Figure 2**, the expression levels of E-cadherin (CDH1) were repressed whereas the expression of N-cadherin (CDH2) and Vimentin (VIM) were enhanced after Id2 depletion. In addition, Id2 knockdown (CL1-0/shID2-284 and CL1-0/shID2-528) significantly increased the proliferation activities as well as the anchorage-dependent and -independent growth capabilities of the CL1-0 cells relative to the control cells (**Figures 3D–F**; *P* < 0.05). Moreover, the migratory and invasive activities of the CL1-0/shID2-528 cells relative to the control cells were significantly increased (**Figures 3G, H**; *P* < 0.05). However, the CL1-0/shID2-284 cells and control cells (CL1-0/shNC) had nearly the same migration abilities but significantly increased invasiveness in the CL1-0/shID2-284 cells (**Figure 3H**; *P* < 0.05). To assess *in vivo* whether Id2 can inhibit the metastasis of lung cancer, mice received tail-vein injections of the CL1-0/shID2-528 cells, and metastatic pulmonary nodule formation on the surface of the lung was examined after 14 weeks. Similar to what we observed *in vitro*, more pulmonary nodules were observed in the mice receiving the CL1-0/shID2-528 cells than in those receiving the CL1-0/shNC control cells (**Figures 3I, J**, 20 ± 4.67 vs. 6.4 ± 1.31 nodules; *P* = 0.05). H&E staining was performed to assess the morphology of the metastatic lung nodules. (**Figure 3J**). According to these results, Id2 is involved in inhibiting metastasis in LADC cells.

### Id2 downstream target-gene identification through RNA sequencing analysis

**Figure 4:**
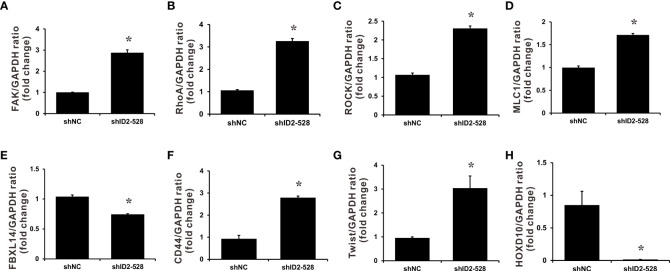
Downstream target genes regulated by Id2 knockdown. mRNA expression levels of **(A)** FAK, **(B)** RhoA, **(C)** ROCK, **(D)** MLC1, **(E)** FBXL14, **(F)** CD44, **(G)** Twist, and **(H)** HOXD10 target genes in Id2-knockdown cells and shNC control cells were measured using qRT-PCR. Internal control: GAPDH. Data (means ± SDs) were collected from three independent experiments. *P < 0.05 versus shNC control.

To characterize how Id2 inhibits the invasion and metastasis of LADC cells, differentially expressed genes between the Id2-knockdown transfectant (CL1-0/shID2-528) and CL1-0/shNC control cells were identified through RNA sequencing analysis. The expression levels of 1,746 genes in total exhibited at least twofold changes between the CL1-0/shID2-528 and CL1-0/shNC control cells. Among them, 1,154 genes were upregulated and 592 were downregulated after Id2 knockdown. The potential Id2-associated molecular mechanisms of differentially expressed genes were assessed using KEGG pathway analysis. **Table 1** lists the five top significant pathways, which included focal adhesion and proteoglycan in cancer pathways. To confirm the data from the RNA sequencing analysis, we first determined the focal adhesion- and proteoglycan-related gene expression levels in Id2-knockdown transfectant (CL1-0/shID2-528) and CL1-0/shNC control cells by using SYBR Green qRT-PCR. As illustrated in **Figure 4** and **Supplementary Table 2**, Id2 knockdown significantly increased the mRNA expression levels of ras homolog family member A (RhoA), focal adhesion kinase (FAK), megalencephalic leukoencephalopathy with subcortical cysts 1 (MLC1), and rho-associated protein kinase (ROCK) (P < 0.05). In addition, Twist family bHLH transcription factor 1 (Twist 1) and CD44, which are involved in the proteoglycan-related pathway, were significantly increased under Id2 knockdown (P < 0.05). However, compared with the control cells, in the knockdown cells, Id2 knockdown could significantly reduce F-box and leucine-rich repeat protein 14 (FBXL14) and homeobox D10 (HOXD10) expression (*P* < 0.05).

**Table 1:** KEGG pathway enrichment analysis.

Module/Term.	Description.	Count.	*P* value.
hsa04510	Focal adhesion	31	0.0002
hsa04512	ECM-receptor interaction	16	0.0007
hsa05205	Proteoglycan in cancer	29	0.0013
hsa04350	TGF-beta signaling pathway	16	0.0024
hsa04151	PI3K-Akt signaling pathway	43	0.0036

Top five significant pathways are ranked based on their *P* value, and terms with *P* < 0.05 are statistically significant.

### LADC survival prediction by using Id2 plus FAK-Twist-gene signature

**Figure 5:**
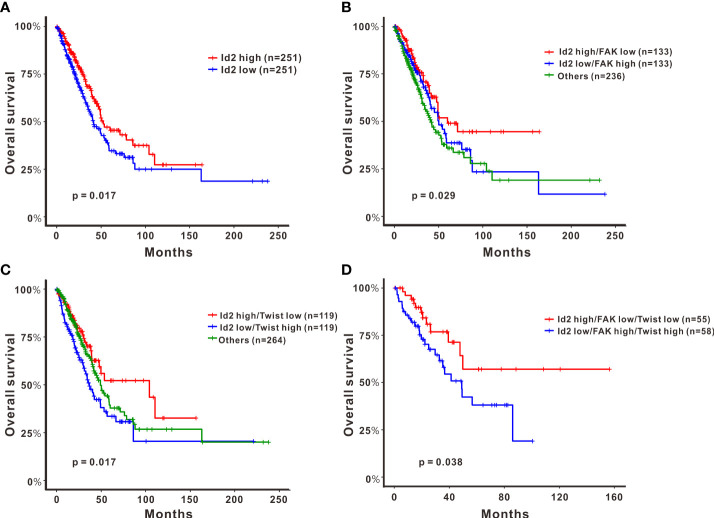
Effects of the expression of Id2 and its target genes on overall survival in patients with LADC. Kaplan–Meier curves and log-rank test results derived for overall survival with respect to the expression of **(A)** Id2, **(B)** Id2 and FAK, **(C)** Id2 and Twist, and **(D)** a combination of the three genes Id2, FAK, and Twist. Significance was reached when *P* < 0.05.

To elucidate how Id2 is clinically relevant to patients with LADC, we further analyzed the genetic data of 502 patients with LADC derived from TCGA. Our analysis (log-rank test) results revealed that the group with highly expressed Id2 had longer overall survival (*P* = 0.017; **Figure 5A**). Additionally, the group with high Id2 expression plus low FAK expression had the longest overall survival (log-rank test, *P* = 0.029; **Figure 5B**). Furthermore, relative to the other groups, in the group with high Id2 expression plus low Twist expression, the observed overall survival was significantly longer (log-rank test, *P* = 0.017; **Figure 5C**). For the high- and low-risk groups with these three gene signatures, the Kaplan–Meier survival curves were separable and revealed a significantly unfavorable survival rate only in the group with both high FAK and high Twist expression and the group with low Id2 expression (log-rank test, *P* = 0.038; **Figure 5D**).

## Discussion

As the most prominent lung cancer type, NSCLC constitutes the prominent cause of deaths related to cancer globally (1). Of patients who have been diagnosed as having NSCLC, nearly 30% have early-stage disease, for which curative surgery is executed. However, within 5 years, NSCLC relapses for up to 40% of patients (39). To find genes that affect the acquisition of metastatic phenotypes in LADC cells, we used a cDNA microarray to screen this model cell line, detecting dozens of genes associated with invasiveness (6). The expression of the Id2 candidate gene had a negative correlation with cell line invasiveness. To date, many studies have investigated the functions of Id proteins in tumorigenesis in various cancers; however, Id2’s function in cancer is still unclear (11). Even though evidence indicates that Id2 exhibits different functions in various cancer types, its function in lung cancer requires further investigation. Accordingly, we executed the study reported herein to identify and characterize the roles of Id2 in LADC progression and metastasis. Our data reveal higher Id2 expression in human LADC CL1-0 cells with low invasiveness than in CL1-5 cells with high invasiveness. This result suggests that Id2 is a putative invasion suppressor in LADC. After the achievement of constitutive Id2 expression in the CL1-5 cells, the invasive and migratory capabilities of these Id2-overexpressing cell clones were significantly reduced relative to those of the parental CL1-5 and mock control cells. In addition, Id2 overexpression could inhibit lung cancer cells’ proliferation and colony formation.

We reduced gene overexpression’s artificial effect on cell physiology and behavior by establishing an Id2-silenced cell line model; we executed this process because of the frequent inactivation of the tumor suppressor genes in lung cancer (40). Our data reveal that Id2 knockdown could significantly increase the CL1-0 cells’ proliferation, migratory, invasive, and colony formation capabilities. Id2-silenced CL1-0 cells also exhibited a mesenchymal-like morphology. Whether Id2 can affect the mesenchymal‐to‐epithelial transition (MET)/epithelial‐to‐mesenchymal transition (EMT) processes in lung cancer cells through Twist regulation requires further investigation; however, our data at least demonstrate that Id2 knockdown increased the mRNA expression of Twist. Furthermore, in the animal model, Id2-silenced cells increased nodule formation *in vivo*. All the aforementioned results are consistent with our prediction that Id2 is an invasion suppressor in LADC. Notably, although two Id2-specific shRNAs significantly reduced Id2 mRNA and protein expression levels, they demonstrated different characteristics in terms of regulation of LADC cell functions. As shown in **Figure 3B**, shID2-528 is more effective than shID2-284 in decreasing the ID2 protein expression in lung cancer cells. shID2-284 could significantly increase colony formation, cell proliferation and invasion in lung cancer; however, it exhibited few effects on cell migratory capabilities. By contrast, shID2-528 significantly affected all the tested functions of lung cancer cells *in vitro* and *in vivo*. This little discrepancy might be due to the different inhibitory efficiency between these two shRNAs, and different levels of ID2 inhibition lead to differences in cellular functions.

To further investigate the correlation of Id2 with clinical outcomes, the gene expression data of 502 patients with LADC derived from TCGA were analyzed. The results indicate that patients with LADC with high Id2 expression levels would have superior clinical outcomes. Our data are consistent with those reported by a previous study that analyzed another database using the Kaplan–Meier plotter (41). Additionally, our results suggest that the Id2 plus FAK-Twist gene signature could predict survival in patients with LADC. Previous study demonstrated that high nuclear expression of ID2 predicts poor prognosis of NSCLC patients with poorly differentiated tumors (22). However, the increased expression of cytoplasmic ID2 was associated with better prognosis in small cell lung cancer patients (28). Whether the different subcellular localizations of ID2 could affect its function in lung cancer cells needs to be investigated in the further studies.

Cancer-related death in humans often occurs because of metastasis. During cancer cell migration, FAK is known to control actin assembly and disassembly and affect cell adhesion dynamics (42). FAK is a cytoplasmic tyrosine kinase that has roles in cell adhesion structure and cytoskeletal remodeling (43,44). The Rho-GTPase pathway plays a critical role in FAK-mediated actin assembly through the stimulation of actin cytoskeleton rearrangement (45). Among the three Rho-GTPases, RhoA can specifically promote the development of stress fiber and focal adhesion. Additionally, FAK, RhoA, and its downstream ROCKs contribute to cell constriction and pseudopodia formation, which are required for cell migration (46,47). Our results show that knockdown of Id2 increased FAK/RhoA expression and the corresponding downstream effectors ROCK1 and MLC. Additional investigations are required to delineate the detailed signaling cascade involved in Id2-mediated actin reorganization.

The bHLH transcription factor Twist is a major EMT regulator, and high Twist expression levels have been associated with cell metastasis, migration, angiogenesis, and drug resistance (48–50). Recent reports have demonstrated that Twist1 was degraded through polyubiquitination mediated by F-box and leucine-rich repeat protein 14 (FBXL14) (51–53). CD44 is a cancer stem cell marker, in addition to being associated with cancer progression and being involved in an EMT-like process in tumor cells (49,54). For instance, stimulation of CD44 in breast cancer cells was demonstrated to activate Twist expression, thus regulating the EMT phenotype through lysyl oxidase activation (55). The present results demonstrate that Id2 knockdown reduced FBXL14 expression and upregulated Twist expression. We propose this regulation might happen partly through CD44 augmentation. Dysregulation of microRNAs is observed in various types of cancers. Furthermore, miR-10b, induced by Twist, has been found to function as an oncogenic microRNA involved in the metastasis and invasion of tumors in various cancers, which have a downstream major mediator, namely the tumor suppressor gene HOXD10 (56–58). Our results signify that upregulation of Twist under Id2 knockdown may lead to HOXD10 reduction and probably block or reduce its tumor suppressor activities. Whether miR-10b is involved in this regulation in lung cancer cells requires further investigation.

## Conclusions

In summary, although Id2’s comprehensive functions in LADC progression have not yet been clarified, the present study demonstrated that Id2 might suppress metastasis in the progression of LADC. We hypothesize that this effect of Id2 occurs at least partially by inhibiting the FAK-related pathway and CD44/Twist axis (**Supplementary Figure 3**). This proposed mechanism of function of Id2 in lung cancer needs to be validated experimentally in further studies. All the efforts in the present study not only extend our knowledge about the functional roles of the Id2 gene in LADC progression and metastasis but also indicate that the Id2 gene is a strong prognostic factor in patients with LADC. An enhanced understanding of Id2-regulated signaling pathways and molecules is critical for developing novel and more effective treatment strategies for future LADC therapies.

## Ethic statement

Animal studies were carried out under protocols approved by the Laboratory Animal Center, National Taiwan University College of Medicine (IACUC number: 20140034).

## Data availability statement

The datasets presented in this study can be found in online repositories. The names of the repository/repositories and accession number(s) can be found below: https://www.ncbi.nlm.nih.gov/PRJNA778932.

## Author contributions

J-TC, Y-LH, Y-CH, and Y-HT performed experiments and collected data. M-HL, C-WW, and C-HL carried out data analysis. S-HP and JJWC provided helpful discussions. S-HP and C-CW designed experiments. JJWC and C-CW drafted the manuscript. Y-HT and C-CW revised the manuscript. All authors read and approved the final manuscript. All authors contributed to the article and approved the submitted version.

## Funding

This study was supported by grants from the Cathay General Hospital, Taiwan, ROC. (99 CGH-FJU-99-02), as well as by grants from the Ministry of Science and Technology, Taiwan, ROC. (MOST 107-2314-B-030-010-MY2 and 109-2314-B-030-010-MY3).

## Acknowledgments

The authors would like to thank Wallace Academic Editing for manuscript English editing.

## Conflict of interest

The authors declare that the research was conducted in the absence of any commercial or financial relationships that could be construed as a potential conflict of interest.

## Publisher’s note

All claims expressed in this article are solely those of the authors and do not necessarily represent those of their affiliated organizations, or those of the publisher, the editors and the reviewers. Any product that may be evaluated in this article, or claim that may be made by its manufacturer, is not guaranteed or endorsed by the publisher.

## Author disclaimer

The funders had no role in study design, data collection and analysis, decision to publish, or preparation of the manuscript.

## Abbreviations

LADC, lung adenocarcinoma; Id2, inhibitor of DNA binding 2; FAK, focal adhesion kinase; NSCLC, Non–small cell lung cancer; bHLH, basic helix–loop–helix; GAPDH, glyceraldehyde-3-phosphate dehydrogenase; KEGG, Kyoto Encyclopedia of Genes and Genomes; RhoA, ras homolog family member A; MLC1, megalencephalic leukoencephalopathy with subcortical cysts 1; ROCK, rho-associated protein kinase; Twist, Twist family bHLH transcription factor; FBXL14, F-box and leucine-rich repeat protein 14; HOXD10, homeobox D10; MET, mesenchymal‐to‐epithelial transition; EMT, epithelial‐to‐mesenchymal transition.
