# Supplementary material for: Id2 exerts tumor suppressor properties in lung cancer through its effects on cancer cell invasion and migration
Source: Front Oncol. 2022 Aug 2;12:801300. doi: 10.3389/fonc.2022.801300 (PMC9379288; doi:10.3389/fonc.2022.801300)
Supplement: Supplementary file 1 [file DataSheet_1.docx]

**Supplementary Materials:**

**Id2 exerts tumor suppressor properties in lung cancer through its effects on cancer cell invasion and migration**

Jian-Ting Chen, Yuan-Ling Hsu, Yi-Chiung Hsu, Yi-Hsin Tseng, Ming-Han Liu, Chia-Wei Weng, Ching-Hao Lin, Szu-Hua Pan, Jeremy J.W. Chen, Chi-Chung Wang

**Correspondence:** Chi-Chung Wang, Ph.D., Graduate Institute of Biomedical and Pharmaceutical Science, Fu Jen Catholic University, No. 510, Zhongzheng Rd., Xinzhuang Dist., New Taipei City, 24205, Taiwan (R.O.C.) Phone: 886-2-29052039, Fax: 886-2-29053415, Email: 075006@mail.fju.edu.tw.

**Supplementary Table 1.** Primers used for SYBR Green real-time reverse transcription-polymerase chain reaction

| **Gene targets** | **Forward primer (5’-3’)** | **Reverse primer (5’-3’)** |
| --- | --- | --- |
| GAPDH | GTCTCCTCTGACTTCAACAGCG | ACCACCCTGTTGCTGTAGCCAA |
| FAK | GAAGCATTGCGGGAACTA | CTCAATGCAGTTTGGAGGTGC |
| RhoA | AAGGACCAGTTCCCAGAGGT | TTCTGGGGTCCACTTTTCTG |
| Rock1 | GAAGCTCGAGAGAAGGCTGA | TTGTCTGCCTCAAATGCTTG |
| MLC1 | CCAAGAAAGCTGCATCGAACCAT | CAGCACATTCCTGATGCCACCT |
| CD44 | AGCAACCAAGAGGCAAGAAA | GTGTGGTTGAAATGGTGCTG |
| FBXL14 | TGTACGGCTGCACCCGAATCAC | CTTCCCCGAGTTCTCACAGTGA |
| Twist | GTCCGCAGTCTTACGAGGAG | TGGAGGACCTGGTAGAGGAA |
| HOXD10 | GCTCCTTCACCACCAACATT | AAATATCCAGGGACGGGAAC |
| ID2 | ATGAAAGCCTTCAGTCCCGT | TTCCATCTTGCTCACCTTCTT |
| TBP | CACGAACCACGGCACTGATT | TTTTCTTGCTGCCAGTCTGGAC |

**Supplementary Table 2.** Genes stimulated or suppressed in CL1-0 cells following *Id2* gene knockdown*

| GenBank accession code | Gene symbol | | Gene name | shID2-528/shNC (Fold change)‡ | | | |  |
| --- | --- | --- | --- | --- | --- | --- | --- | --- |
|  |  |  |  | RNA Sequencing | | Real-time RT-PCR | |  |
| Stimulated genes |  | |  |  | |  | |  |
| NM_002345 | LUM | Lumican | | | 8.76 | | 9.02 | |
| NM_000474 | Twist1 | Twist Family BHLH Transcription Factor 1 | | | 5.94 | | 3.21 | |
| NM_001376 | MLC1 | Megalencephalic leukoencephalopathy with subcortical cysts 1 | | | 2.55 | | 1.72 | |
| NM_001385 | ANG | Angiogenin | | | 2.24 | | 3.57 | |
| NM_000610 | CD44 | CD44 Molecule | | | 2.09 | | 3.03 | |
| NM_005406 | ROCK | Rho Associated Protein Kinase | | | 1.58 | | 2.16 | |
| NM_001313 | RhoA | Ras Homolog Family Member A | | | 1.44 | | 3.09 | |
| NM_001387 | FAK | Focal adhesion kinase | | | 0.71 | | 2.88 | |
| Suppressed genes |  |  | | |  | |  | |
| NM_002148 | HOXD10 | Homeobox D10 | | | 0.14 | | 0.01 | |
| NM_152441 | FBXL14 | F-box and leucine-rich repeat protein 14 | | | 0.72 | | 0.72 | |

*The differentially expressed genes related to focal adhesion and proteoglycan-related signaling, which were identified by RNA Sequencing, were validated by SYBR Green real-time RT-PCR.

‡ shID2-528 = CL1-0 cells transfected with Id2 shRNA; shNC = CL1-0 cells transfected with vector control. RT-PCR = reverse transcription-polymerase chain reaction.

**Supplementary Figure 1.** Overexpression of Id2 decreases the filopodia numbers in CL1-5 cells. Quantification of filopodia numbers in Id2-overexpressing (ID2) or vector-transfected (Mock) CL1-5 cells. Filopodia numbers of Mock (n=74) and ID2 (n=92) cells were counted and calculated. Columns, means of average filopodia number per cell; bars, SD. *** P＜0.001.


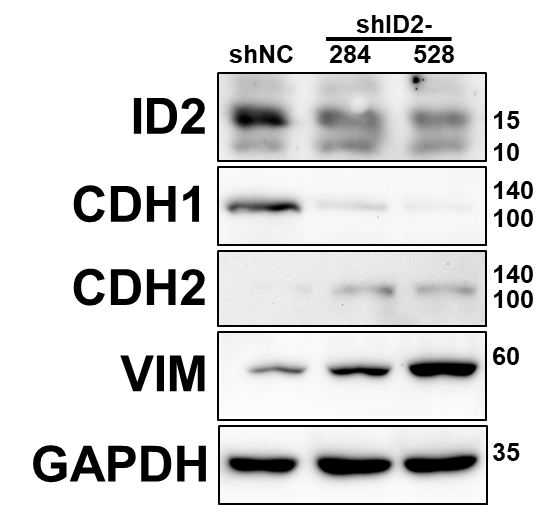


**Supplementary Figure 2.** Expression levels of EMT markers in Id2-knockdown CL1-0 cells. Protein levels of EMT markers in Id2-knockdown CL1-0 cells were assayed by immunoblotting. Protein lysate from shID2-284, shID2-528 and shNC control cells were developed by SDS-PAGE and used to perform immunoblotting assay. Internal or loading control: GAPDH.


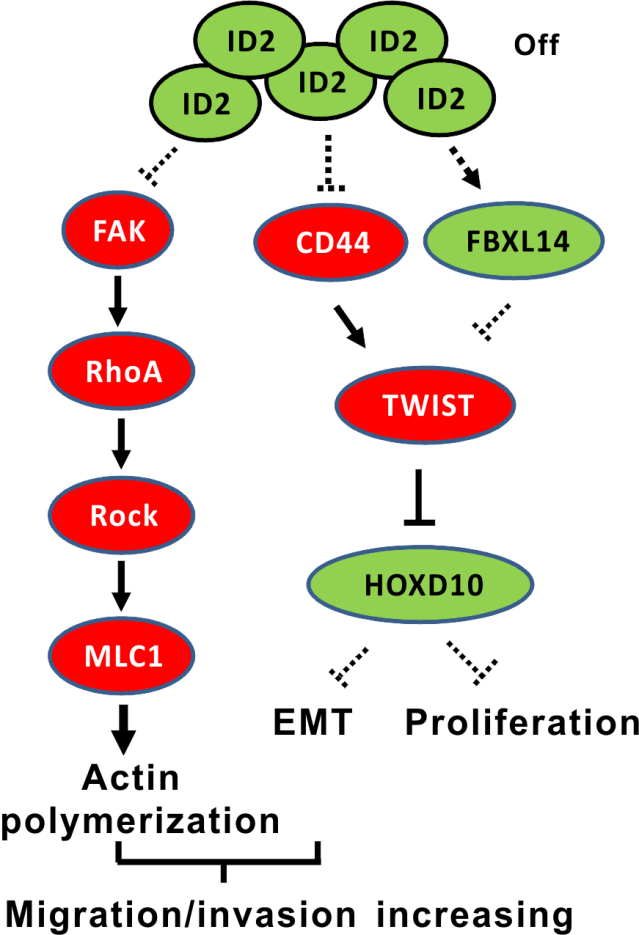


**Supplementary Figure 3.** Proposed model of Id2 knockdown–mediated cancer cell aggressiveness promotion. In Id2-knockdown LADC cells, the expression levels of FAK signaling–related genes were increased, yielding enhanced cell invasive and migratory capabilities. Additionally, CD44/Twist gene expression levels were upregulated under Id2 knockdown and promoted lung cancer cell proliferation and EMT processes.


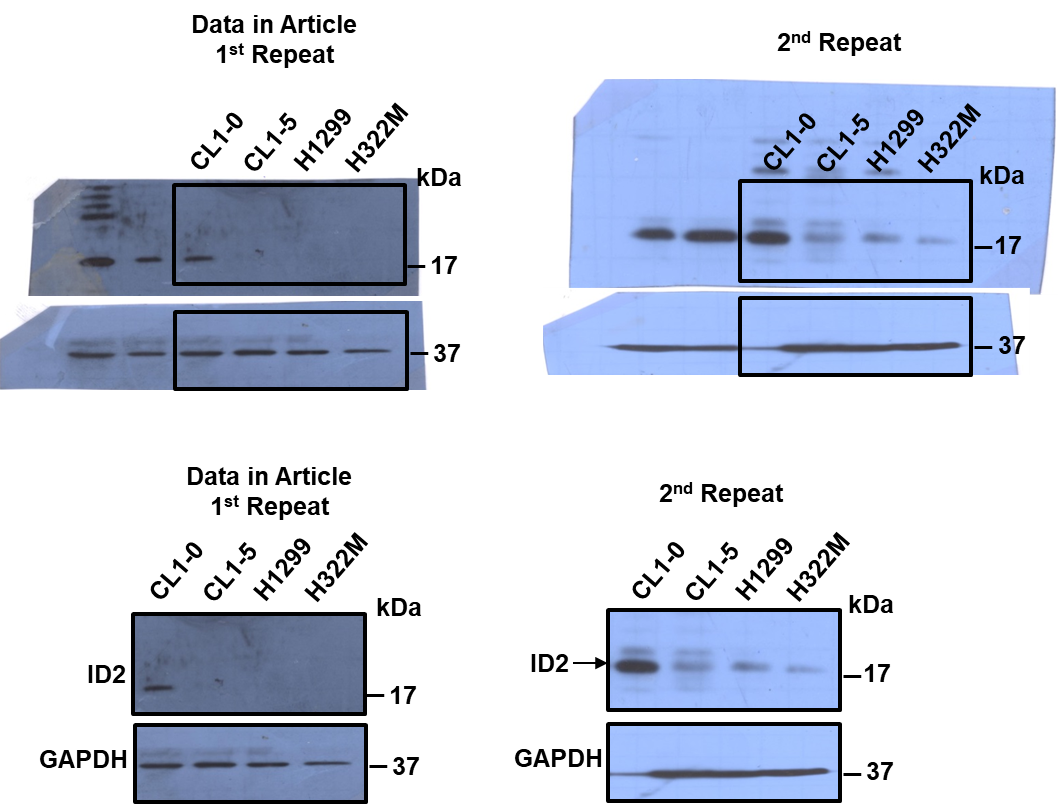


**Supplementary Figure 4.** The original results of blots from Figure 1C.


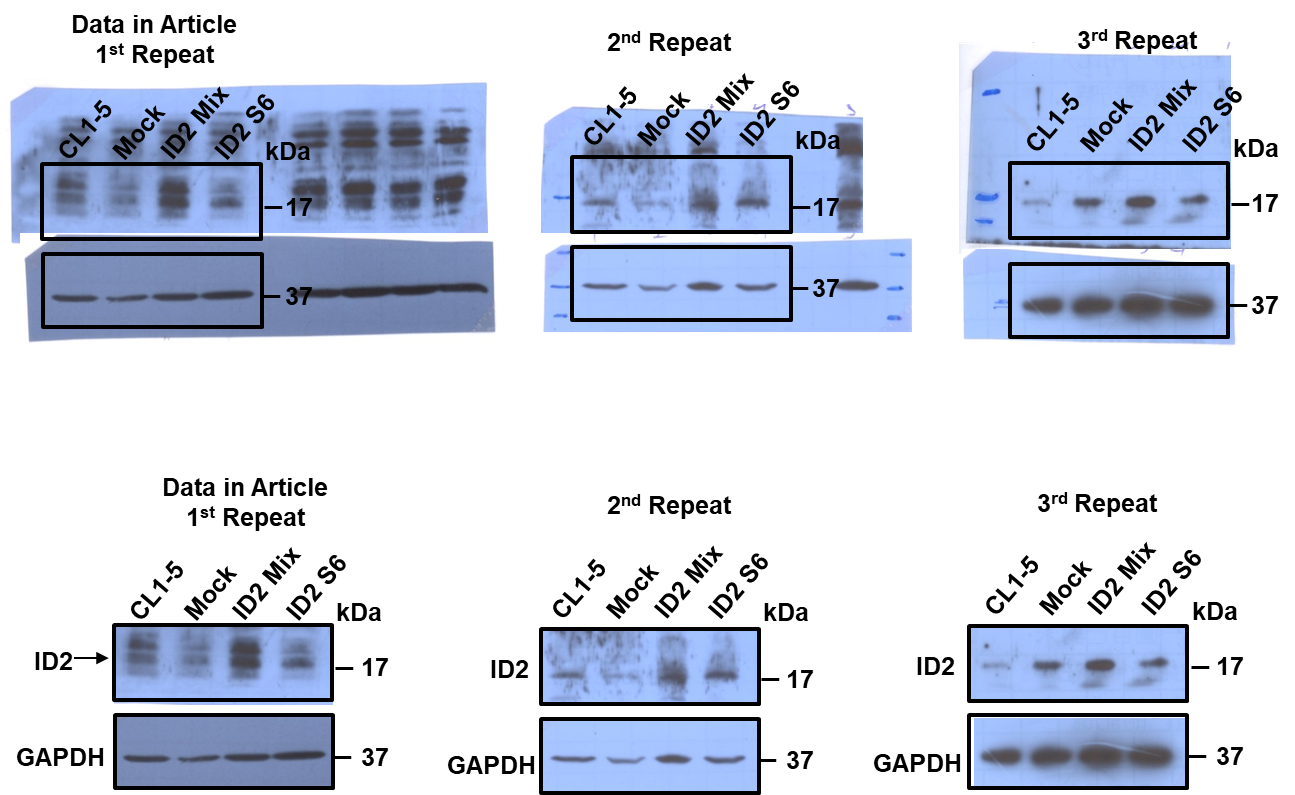


**Supplementary Figure 5.** The original results of blots from Figure 2B.


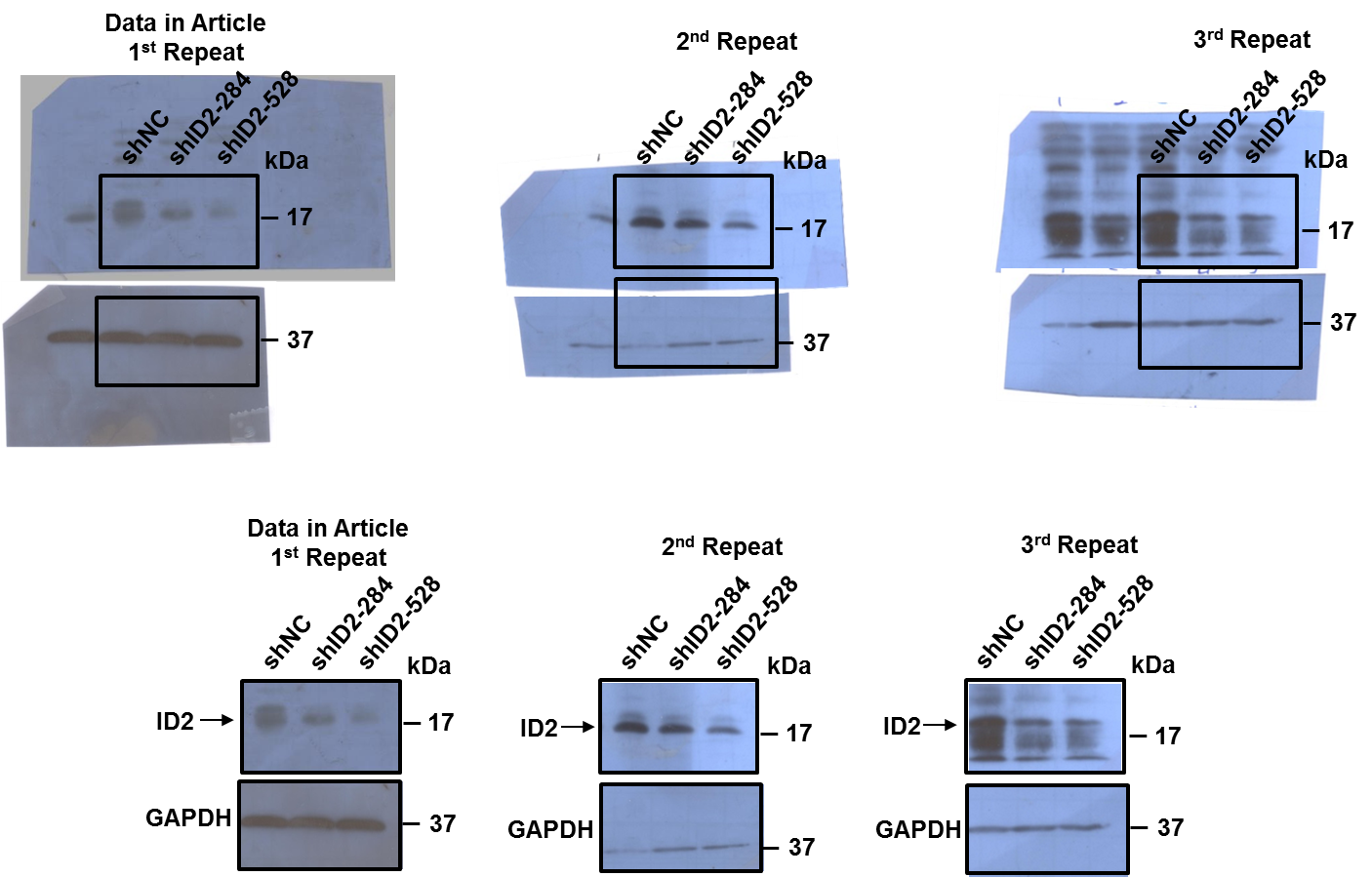


**Supplementary Figure 6**. The original results of blots from Figure 3B.
